# Evaluation of Outcomes from Pedicle Screw Fixation Before and After Laminectomy: Short title: Outcomes of Pedicle Screw Fixation Before and After Laminectomy

**DOI:** 10.31661/gmj.v15i.3856

**Published:** 2026-06-12

**Authors:** Vahid Sadeghi, Seyed Taher Mousavi, Mohammad Shimia

**Affiliations:** ^1^ Department of Neurosurgery, Tabriz University of Medical Sciences, Tabriz, Iran; ^2^ Department of Neurosurgery, Faculty of Medicine, Tabriz University of Medical Sciences, Tabriz, Iran

**Keywords:** Pedicle Screw Fixation, Laminectomy, Spinal Stabilization

## Abstract

**Background:**

Pedicle screw fixation is a standard technique for spinal stabilization in patients undergoing laminectomy for decompression. This study aims to compare the outcomes of pedicle screw fixation performed before versus after laminectomy, focusing on its effects on spinal stability, recovery outcomes, and complication rates.

**Materials and Methods:**

This retrospective cohort study, conducted from July 2017 to July 2020 at the Neurosurgery Departments of Imam Reza and Shohada Hospitals in Tabriz, Iran, assessed outcomes of two approaches to pedicle screw fixation in 104 patients undergoing laminectomy for degenerative spinal conditions. The patients were equally divided into two groups of 52: one group receiving pedicle screw fixation before laminectomy, and the other following laminectomy. Key intraoperative parameters—such as blood loss, operative time, and screw placement accuracy—were evaluated and compared between groups. Data were analyzed using SPSS software, employing Independent Samples T-Tests and Chi-square tests, with statistical significance defined at P0.05. This retrospective cohort study compares two surgical sequences—pedicle screw fixation before versus after laminectomy—in patients undergoing lumbar decompression, aiming to evaluate intraoperative efficiency and short-term outcomes.

**Results:**

The laminectomy-first group had a lower infection rate (5.76% vs. 9.61%) and required fewer blood transfusions (96.1% vs. 90.3%) than the screw-first group, though these differences were not significant (P0.05). Blood loss and drain output were significantly higher in the laminectomy-first group (P0.05), but surgery duration was shorter (P=0.01), with similar hospital stays between groups. The laminectomy-first approach also required fewer screw path corrections and fluoroscopy uses, indicating greater intraoperative efficiency (P=0.001).

**Conclusion:**

This study demonstrates that pedicle screw fixation after laminectomy demonstrated greater intraoperative efficiency with fewer adjustments and reduced fluoroscopy use. However, due to the retrospective nature of the study and lack of systematic documentation of baseline stability and symptom dominance, future prospective or randomized controlled trials are needed to confirm these findings and guide evidence-based surgical decision-making.

## Introduction

Spinal stabilization, particularly through pedicle screw fixation, is integral to the management of complex spinal disorders, including degenerative diseases, traumatic injuries, and oncologic conditions[1][2]. The pedicle screw system, which has been extensively validated for its biomechanical efficacy, enhances vertebral stability by securing adjacent vertebrae, promoting vertebral fusion, and ultimately restoring alignment and stability to the spine [3]. As such, pedicle screw fixation is commonly employed to stabilize the spine in patients undergoing laminectomy—a decompressive surgical technique frequently indicated for relieving nerve compression caused by spinal stenosis, herniated discs, or tumors [4]. The integration of pedicle screw fixation in these surgical procedures has demonstrated significant advantages in improving patient outcomes, as highlighted in clinical studies [5]. Du et al. (2017) concluded that patients undergoing lumbar lateral interbody fusion with unilateral pedicle screw fixation for adult spinal deformity treatment experienced significant reductions in pain and favorable radiographic outcomes [6].

While pedicle screw fixation is widely used to stabilize the spine during laminectomy, the impact of timing (pre-laminectomy vs. post-laminectomy fixation) on surgical outcomes remains understudied. Most existing evidence focuses on biomechanical properties of fixation techniques (e.g., screw pullout strength after laminectomy [7] or animal models of spinal stability [8]., rather than direct comparisons of pre- and post-laminectomy fixation. To date, clinical studies directly evaluating the effects of fixation timing are limited, with small sample sizes and conflicting results [9][10]. This gap highlights the need for larger comparative studies to guide surgical decision-making.

Pre-laminectomy fixation, for instance, has been associated with improved intraoperative stability, which may mitigate the risks of vertebral shifting or collapse during decompression [11][8]. Studies reported that pedicle screws placed before laminectomy significantly enhanced vertebral stability, facilitating more secure decompression and reducing the likelihood of complications arising from spinal instability [12][13]. On the other hand, post-laminectomy fixation offers the potential for enhanced surgical precision. Yan et al. (2024) found that the post-laminectomy fixation allowed for enhanced surgical precision by providing better access to the spinal cord during the emergency intervention for the hematoma. This approach facilitated more accurate decompression and timely relief of spinal cord pressure, contributing to the patient's recovery [14]. This flexibility is particularly advantageous in cases requiring intricate decompression, as the absence of pre-positioned screws can improve access to affected neural structures.

Despite these insights, direct comparisons of outcomes between pre- and post-laminectomy fixation remain limited, primarily due to small sample sizes and single-center study designs. As a result, the generalizability of the findings is restricted, and no consensus has been established regarding the optimal timing for screw placement. This gap in robust comparative evidence highlights the need for further research into whether the timing of pedicle screw fixation in relation to laminectomy impacts clinical and functional outcomes. Given the mixed results, a larger, comparative study is necessary to evaluate whether the timing influences factors such as postoperative pain, recovery time, spinal stability, and complication rates. This retrospective study aims to address this gap by systematically assessing outcomes of pedicle screw fixation performed before versus after laminectomy across a large patient cohort.

## Materials and Methods

This retrospective cohort study was conducted to evaluate the outcomes of two different approaches to pedicle screw fixation in patients undergoing laminectomy for degenerative spinal conditions... The study comprised 208 consecutive patients who underwent pedicle screw fixation either before or after laminectomy for degenerative spinal conditions at Imam Reza and Shohada Hospitals (Tabriz, Iran) between July 2017 and July 2020. However, these decisions were not systematically documented, and baseline data on spinal instability (e.g., spondylolisthesis grade), radiographic parameters (e.g., sagittal balance), or symptom dominance (axial versus leg pain) were not collected [15] . Retrospective data collection included all eligible cases within this period, without arbitrary group allocation. The equal distribution of patients into two groups was based on convenience sampling and statistical power considerations rather than natural clinical variability. While this enhances comparative power, it may not reflect real-world surgical decision-making patterns.

Patients were selected based on the following inclusion criteria: age between 15 and 60 years, degenerative disc changes at the L3/L4, L4/L5, or L5/S1 levels, and completion of the informed consent form. The choice of surgical sequence (pedicle screw fixation before or after laminectomy) was determined by the operating surgeon’s discretion, guided by intraoperative assessment of spinal stability, symptomatology, and anatomical factors in accordance with established guidelines (e.g., World Federation of Neurosurgical Societies Spine Committee recommendations). For example, pre-laminectomy fixation was prioritized in cases of unstable spondylolisthesis or sagittal imbalance, while laminectomy-first approaches were favored for decompression-dominant cases with no instability. Retrospective data did not capture detailed rationales for individual decisions, which is a limitation of this study.

The study compared two groups: one underwent pedicle screw fixation before laminectomy (pre-laminectomy group) and the other after laminectomy (post-laminectomy group). Key intraoperative variables such as blood loss, operative time, and screw placement accuracy were compared between the two groups. Additionally, the study utilized a stepwise method for screw placement, including identification of anatomical landmarks, drilling, and fluoroscopic guidance to ensure proper screw insertion. Data were analyzed using SPSS software (version 20) with descriptive statistics and inferential tests, including the Independent Samples T-Test, Kolmogorov-Smirnov test for normality, and Chi-square tests for categorical data. The significance level was set at P<0.05.

### Ethical Consideration

All procedures were approved by the Ethics Committee of Tabriz University of Medical Sciences (IR.TBZMED.REC.1398.1172).

## Results

**Table:** Table1. Demographic Characteristics of Patients

**Group**		**Laminectomy, then Pedicle Screw (n=52) **	**Pedicle Screw, then Laminectomy (n=52) **	**Total (n=104)**	**P-Value**
Variables		**laminectomy followed by pedicle screw fixation **	**Pedicle screw fixation followed by laminectomy **	**Total**	**P-value**
**Gender**	**Male (%)**	40 (76.9%)	37 (72.2%)	77 (74.1%)	0.567
	**Female (%)**	12 (23.1%)	15 (28.8%)	27 (25.9%)	
**Age (Mean ± SD) **		39.90 ± 5.89	43.0 ± 3.30		0.07
**BMI (Mean ± SD) **		28.7 ± 4.3	26.3 ± 6.3		0.68

**Table:** Table2. Postoperative Complications and Transfusion Rates

Group	**Postoperative Infection (%) **	**P-Value**	**Blood Transfusion (Units) **	**P-Value**
laminectomy followed by pedicle screw fixation	3 (5.76%)	0.073	58 units in 50 patients (96.1%)	0.063
Pedicle screw fixation followed by laminectomy	5 (9.61%)		51 units in 47 patients (90.3%)	

**Table:** Table3. Surgical Parameters and Outcomes

**Group**	**Intraoperative Blood Loss (mL ± SD) **	**P-Value**	**Drain Output Day 1 (mL ± SD) **	**P-Value**	**Drain Output Day 2 (mL ± SD) **	**P-Value**	**Surgery Duration (hours ± SD) **	**P-Value**	**Length of Stay (days) **	**P-Value**
**laminectomy followed by pedicle screw fixation **	726.0 ± 57	0.035	184.3 ± 18.6	0.02	64.6 ± 6.4	0.58	2.8 ± 0.6	0.01	3-7 days (51.9%)	0.76
P **edicle screw fixation followed by laminectomy **	587.0 ± 62		167.6 ± 16.8		68.7 ± 5.0		3.5 ± 0.0		3-7 days (57.7%)	

**Table:** Table4. Comparison of Pre- and Postoperative Laboratory Values and Intraoperative Parameters Between Surgical Techniques

**Parameter**	**Time Point**	**Group**	**Mean ± SD**	**p-Value**
	Before Surgery	laminectomy followed by pedicle screw fixation	14.4 ± 1.68	0.466
		Pedicle screw fixation followed by laminectomy	14.2 ± 1.89	
**Hemoglobin Levels (g/dL)**	After Surgery (Day 1)	laminectomy followed by pedicle screw fixation	11.8 ± 1.59	0.576
		Pedicle screw fixation followed by laminectomy	12.6 ± 1.79	
	Day 2 After Surgery	laminectomy followed by pedicle screw fixation	12.7 ± 1.57	0.599
		Pedicle screw fixation followed by laminectomy	12.8 ± 1.77	
	Before Surgery	laminectomy followed by pedicle screw fixation	39.1 ± 5.02	0.763
		Pedicle screw fixation followed by laminectomy	39.4 ± 5.78	
**Hematocrit Levels (%)**	After Surgery	laminectomy followed by pedicle screw fixation	37.6 ± 4.54	0.694
		Pedicle screw fixation followed by laminectomy	38.1 ± 5.56	
	Day 2 After Surgery	laminectomy followed by pedicle screw fixation	38.5 ± 4.23	0.587
		Pedicle screw fixation followed by laminectomy	38.4 ± 5.98	
	Screw Path Correction	laminectomy followed by pedicle screw fixation	5 (times used)	0.001
**Screw Placement and Procedures**		Pedicle screw fixation followed by laminectomy	88 (times used)	
	Fluoroscopy Use	laminectomy followed by pedicle screw fixation	10 (times used)	0.001
		Pedicle screw fixation followed by laminectomy	98 (times used)	

Table-1 presents the demographic characteristics of patients undergoing two surgical approaches: "laminectomy followed by pedicle screw fixation" and "pedicle screw fixation followed by laminectomy." Both groups included 52 patients, with a comparable gender distribution across groups (P=0.567), with 76.9% male in the laminectomy-first group and 72.2% in the screw-first group. Although there was no significant difference in age (mean ± SD of 39.90 ± 5.89 for laminectomy first and 43.0 ± 3.30 for screw-first, P=0.07) or BMI (28.7 ± 4.3 and 26.3 ± 6.3, respectively, P=0.68), these values provide a balanced baseline for comparing surgical outcomes and postoperative complications between the techniques.

Table-2 summarizes postoperative infection and transfusion rates between surgical groups. Infection occurred in 5.76% of patients with the laminectomy-first approach and 9.61% with the screw-first approach, without significant difference (P=0.073). Transfusion needs were similar, with 96.1% of the laminectomy-first group and 90.3% of the screw-first group requiring blood transfusions (P=0.063). Table-3 showed that Laminectomy followed by screw fixation had significantly higher blood loss (726.0 ± 57 mL vs. 587.0 ± 62 mL, P=0.035) and greater drain output on day 1 (P=0.02). Surgery duration was shorter for this group (2.8 ± 0.6 hours vs. 3.5 hours, P=0.01), while length of stay was similar across both groups (P=0.76). Table-4 showed that Both groups had similar hemoglobin and hematocrit levels before and after surgery (P>0.05). However, the "Laminectomy first" group required significantly fewer screw path corrections (5 vs. 88) and less fluoroscopy (10 vs. 98), indicating greater intraoperative efficiency (P=0.001).

## Discussion

Our findings align closely with those reported by ElMesallamy WA et al.who evaluated pre- versus post-decompression screw fixation in the context of posterior lumbar interbody fusion (PLIF)[16]. They observed improved visualization and screw accuracy when fixation followed decompression. Although our cohort primarily involved decompression-dominant cases without fusion, both studies showed reduced fluoroscopy use and fewer screw adjustments in the post-laminectomy group, supporting its technical advantages in non-instability scenarios

In this study, we compared two common surgical approaches for spine surgery—laminectomy followed by pedicle screw fixation and pedicle screw fixation followed by laminectomy—focusing on postoperative infection rates, blood transfusion needs, surgical outcomes, and intraoperative parameters. Our results showed comparable infection and transfusion rates between groups, with the laminectomy-first approach demonstrating reduced surgery duration and fluoroscopy use, albeit at the cost of increased blood loss. These findings align closely with ElMesallamy WA et al., who similarly evaluated pedicle screw fixation timing in the context of posterior lumbar interbody fusion (PLIF) [16]. Regarding postoperative infections and transfusion rates, our results showed no significant difference between the two groups. The infection rate in the laminectomy-first group was 5.76%, while the screw-first group had a higher infection rate of 9.61%. This difference, though not statistically significant, may point to a trend that warrants further investigation. Similarly, both groups had high transfusion rates, with 96.1% of the laminectomy-first group and 90.3% of the screw-first group requiring blood transfusions, but again, this difference was not significant. These results are consistent with other studies where transfusion requirements were observed in a high proportion of patients undergoing complex spine surgeries due to significant intraoperative blood loss [17][18].

Notably, ElMesallamy WA et al. directly compared pre- vs. PLIF, reporting improved intraoperative visualization and screw accuracy when fixation followed decompression [16].While their focus on fusion differs from our decompression-dominant cohort, the shared reduction in fluoroscopy use and screw adjustments supports the broader applicability of laminectomy-first fixation in non-instability cases. However, as noted by both studies, the retrospective design and lack of baseline stability/symptom data limit definitive conclusions. Future prospective studies should stratify outcomes based on spinal stability and adherence to clinical guidelines (e.g., WFNS Statements 1-6) to clarify indications for each approach.

Although transfusions are a common necessity, the small differences in infection and transfusion rates between the groups suggest that both techniques are similarly effective in managing these postoperative complications.

In terms of surgical parameters, the "laminectomy-first" group experienced significantly higher blood loss and drain output on Day 1, despite having a shorter surgery duration. The increased blood loss in the laminectomy-first group could be attributed to the extensive soft tissue dissection required for laminectomy, which is known to result in higher bleeding during the procedure [17]. However, the shorter surgery duration in this group suggests that, while there is more blood loss, the procedure is technically more efficient, likely due to the sequential approach of first performing laminectomy, which may allow for better visualization of the spine before screw placement. This aligns with previous research where laminectomy-first approaches were reported to reduce surgery time [19]. Length of hospital stay did not differ significantly between the two groups, which is consistent with studies that show hospital stay duration may be influenced by multiple factors such as post-surgical care protocols, rather than the specific surgical technique used [19].

Finally, in evaluating intraoperative efficiency, the laminectomy followed by the pedicle screw fixation group required significantly fewer screw path corrections and less fluoroscopy, suggesting greater intraoperative accuracy and efficiency in screw placement. This reduction in screw path correction and fluoroscopy usage reflects improved precision in the surgical technique, as it has been shown that laminectomy-first approaches allow for better visualization of anatomical landmarks, thereby reducing the need for intraoperative adjustments and radiation exposure [20]. Less reliance on fluoroscopy not only minimizes radiation exposure to both patients and the surgical team but also contributes to a safer surgical environment [21]. These findings are consistent with the research of Guzey et al. (2019), which found that the laminectomy-first technique improves accuracy and reduces radiation exposure compared to screw-first approaches [20].

## Conclusion

The clinical relevance of these findings is contingent on adherence to evidence-based guidelines. As highlighted by the World Federation of Neurosurgical Societies Spine Committee (Statement 2), patients with lumbar stenosis and stable spondylolisthesis should undergo decompression alone, without fusion. The inclusion of patients requiring fusion in our cohort suggests that either instability was present (e.g., unstable spondylolisthesis, sagittal imbalance) or surgeons deviated from established protocols. This limitation underscores the need for prospective studies that stratify outcomes based on baseline stability and symptom dominance, ensuring alignment with clinical guidelines These factors may contribute to better patient safety and resource management in clinical settings, supporting the clinical relevance of this approach for spine surgeries. However, further studies with larger sample sizes are needed to fully explore the long-term benefits of each technique, particularly in terms of recovery and cost-effectiveness.

## Conflict of Interest

The authors declare no conflict of interest.

## AI Disclosure Statement

During the preparation of this manuscript, the authors used ChatGPT, OpenAI company for language editing, grammar improvement, and liboberry.com for reference management. After its use, the authors thoroughly reviewed, verified, and revised all AI-assisted content to ensure accuracy and originality. The authors take full responsibility for the integrity and final content of the published article.

## References

[R1] Kapoen C, Liu Y, Bloemers FW, Deunk J (2020). Pedicle screw fixation of thoracolumbar fractures: conventional
short segment versus short segment with intermediate screws at the fracture
level—a systematic review and meta-analysis. European Spine Journal.

[R2] Alpizar-Aguirre A, Cabrera-Aldana E, Rosales-Olivares L, Zárate-Kalfópulos B, Gómez-Crespo S, Reyes-Sánchez A (2017). A new technique of pedicle screw placement with the use of
sequential multilevel navigation templates based on patient-specific 3D CT
reconstruction model: applicability in spine deformity. Acta ortopédica mexicana.

[R3] Ishii K, Funao H, Isogai N, Saito T, Arizono T, Hoshino M (2022). The history and development of the percutaneous pedicle screw
(PPS) system. Medicina.

[R4] Liu Z, Zheng J-H, Yuan N, Miao J (2023). Comparison of the clinical effects of lamina replantation and
screw fixation after laminectomy in the treatment of intraspinal tumours. Journal of Orthopaedic Surgery and Research.

[R5] Matsukawa K, Yato Y, Imabayashi H (2020). Impact of screw diameter and length on pedicle screw fixation
strength in osteoporotic vertebrae: a finite element analysis. Asian Spine Journal.

[R6] Du JY, Kiely PD, Al Maaieh, Aichmair A, Huang RC (2017). Lateral lumbar interbody fusion with unilateral pedicle screw
fixation for the treatment of adjacent segment disease: a preliminary report. Journal of Spine Surgery.

[R7] Kwak D-S, Shin J-H, Cho H-J, Chang HG, Park MS, Kim I-S (2018). Fixation strength of pedicle and cortical lumbar vertebral screws
after laminectomy: a cadaver study. Journal of Neurological Surgery Part A: Central European Neurosurgery.

[R8] Meij BP, Suwankong N, Van der, Hazewinkel HA (2007). Biomechanical flexion–extension forces in normal canine
lumbosacral cadaver specimens before and after dorsal laminectomy–discectomy
and pedicle screw–rod fixation. Veterinary surgery.

[R9] Bai J-y, Zhang W, An J-l, Sun Y-p, Ding W-y, Shen Y (2016). True anteroposterior view pedicle screw insertion technique. Therapeutics and clinical risk management.

[R10] Peene L, Le Cacheux, Sauter AR, Joshi GP, Beloeil H, Collaborators PWG (2021). Pain management after laminectomy: a systematic review and
procedure-specific post-operative pain management (prospect) recommendations. European Spine Journal.

[R11] Levy HA, Potes MDA, Nassr AN, Freedman BA, Sebastian AS (2024). Biomechanical analysis of lumbar decompression technique and the
effect on spinal instability: a narrative review. AME Medical Journal.

[R12] Duan Y, Ma J, Miao S, Zhang J, Deng J, Wu H (2021). Comparison of Total Laminectomy and Pedicle Screw Internal
Fixation with Ultrasonic- and Microscopic-Assisted Laminectomy Replantation
for Tumors of the Lumbar Spinal Canal: A Retrospective Study of 60 Cases
from a Single Center. Med Sci Monit.

[R13] Morimoto D, Isu T, Kim K, Sugawara A, Matsumoto R, Isobe M (2012). Microsurgical medial fenestration with an ultrasonic bone curette
for lumbar foraminal stenosis. Journal of Nippon Medical School.

[R14] Yan RZ, Chen C, Lin CR, Wei YH, Guo ZJ, Li YK (2024). Delayed neurological dysfunction following posterior laminectomy
with lateral mass screw fixation: A case report and review of literature. World J Clin Cases.

[R15] Costa F, Anania CD, Zileli M, Servadei F, Fornari M (2020). Lumbar spinal stenosis: introduction to the world federation of
neurosurgical societies (WFNS) spine committee recommendations. World Neurosurgery: X.

[R16] ElMesallamy WAA, Kamel AAF, Elbanna M, Haggag A, AlBakry A (2022). Lumbar Pedicle Screws Fixation before versus after Posterior
Lumbar Interbody Fusion: A Comparative Study and Technical Insight. Advanced Spine Journal.

[R17] Shakeri M, Valaie M, Mirzaei F, Hariri R, Jangholi E, Aeinfar K (2021). Efficacy of Topical Tranexamic Acid in Reducing Blood Loss after
Laminectomy and Posterolateral Fusion of the Spine: A Randomized,
Double-Blinded, Placebo-Controlled Clinical Trial. Journal of Orthopedic and Spine Trauma.

[R18] Erfani M, Ali M, Nooraee M, Momenzadeh M (2020). Posterior Segmental Instrumentation and Fusion of Thoracic and
Lumbar Vertebrae Fractures (Short-Term Outcome). Iranian Journal of Orthopedic Surgery.

[R19] Shin S-R, Lee S-S, Kim J-H, Jung J-H, Lee S-K, Lee G-J (2020). Thoracolumbar burst fractures in patients with neurological
deficit: Anterior approach versus posterior percutaneous fixation with
laminotomy. Journal of Clinical Neuroscience.

[R20] Guzey FK, Emel E, Seyithanoglu MH, Bas NS, Ozkan N, Sel B (2006). Accuracy of pedicle screw placement for upper and middle thoracic
pathologies without coronal plane spinal deformity using conventional
methods. Clinical Spine Surgery.

[R21] Lee SE, Chung CK, Jahng T-A, Kim H-J (2013). Long-term outcome of laminectomy for cervical ossification of the
posterior longitudinal ligament. Journal of Neurosurgery: Spine.

